# Cross-Instrument Comparison of MapCam and OVIRS on OSIRIS-REx

**DOI:** 10.1007/s11214-022-00873-8

**Published:** 2022-02-28

**Authors:** D. R. Golish, A. A. Simon, D. C. Reuter, S. Ferrone, B. E. Clark, J.-Y. Li, D. N. DellaGiustina, C. Drouet d’Aubigny, B. Rizk, D. S. Lauretta

**Affiliations:** 1 Lunar and Planetary Laboratory, University of Arizona, Tucson, AZ USA; 2 NASA Goddard Space Flight Center, Greenbelt, MD USA; 3 Department of Physics and Astronomy, Ithaca College, Ithaca, NY USA; 4 Planetary Science Institute, Tucson, AZ USA

**Keywords:** Instrumentation, Data reduction techniques, Asteroids, OSIRIS-REx, (101955) Bennu

## Abstract

Two of the instruments onboard the OSIRIS-REx spacecraft, the MapCam color imager and the OVIRS visible and infrared spectrometer, observed the surface of asteroid (101955) Bennu in partially overlapping wavelengths. Significant scientific advances have been enabled by using data from these two instruments in tandem, but a robust statistical understanding of their relationship is needed for future analyses to cross-compare their data as accurately and sensitively as possible. Here we present a cross-instrument comparison of data acquired by MapCam and OVIRS, including methods and results for all global and site-specific observation campaigns in which both instruments were active. In our analysis, we consider both the absolute radiometric offset and the relative (normalized) variation between the two instruments; we find that both depend strongly on the photometric and instrumental conditions during the observation. The two instruments have a large absolute offset (>15%) due to their independent radiometric calibrations. However, they are very consistent (relative offset as low as 1%) when each instrument’s response is normalized at a single wavelength, particularly at low phase angles where shadows on Bennu’s rough surface are minimized. We recommend using the global datasets acquired at 12:30 pm local solar time for cross-comparisons; data acquired at higher phase angles have larger uncertainties.

## Introduction

The Origins, Spectral Interpretation, Resource Identification, and Security–Regolith Explorer (OSIRIS-REx) spacecraft (Lauretta et al. 2021, 2017) observed the surface of asteroid (101955) Bennu, a B-type near-Earth asteroid (Clark et al. 2011; Hergenrother et al. 2013; Lauretta et al. 2019a), for approximately two years before sampling regolith from its surface on 2020 October 20. Though the observations taken during those two years were primarily driven by the need to identify a safe and sampleable surface location, they also provided a tremendous dataset for scientific analysis of the asteroid and scientific context for the sample. These data revealed Bennu to have a diverse surface with macroscopic heterogeneity in albedo (Golish et al. 2021c; Lauretta et al. 2019a), color (DellaGiustina et al. 2020), composition (Simon et al. 2020b), photometric response (Golish et al. 2021b; Li et al. 2021; Zou et al. 2021), physical structure (Rozitis et al. 2020; Scheeres et al. 2020), and texture (Bennett et al. 2021; Walsh et al. 2019). Some of these characteristics are enhanced by Bennu’s dynamic history, which includes relatively recent surface changes due to mass movement (Jawin et al. 2020), thermal fracturing (Molaro et al. 2020), impacts (Ballouz et al. 2020), and ongoing particle ejections (Hergenrother et al. 2020; Lauretta et al. 2019b). The MapCam imager of the OSIRIS-REx Camera Suite (OCAMS; Rizk et al. 2018) and the OSIRIS-REx Visible and Infrared Spectrometer (OVIRS; Reuter et al. 2018) provided the data underlying many of these discoveries.

MapCam imaged Bennu’s surface in visible (VIS) wavelengths also observed by OVIRS, providing the opportunity for a direct comparison. The two instruments were designed as complements to each other, with MapCam providing broadband spectrophotometric data at high spatial resolution and OVIRS providing high spectral resolution, that extends into the near-infrared (NIR), with coarse spatial scales. Several studies performed during Bennu’s proximity operations used the instruments’ complementary designs to strengthen their analyses (e.g., DellaGiustina et al. 2021; Kaplan et al. 2020). Though both instruments went through extensive ground and in-flight calibration campaigns (Golish et al. 2020; Rizk et al. 2018; Simon et al. 2018, 2021), those calibrations have independent uncertainties, and no formal attempt has previously cross-calibrated the instruments. Moreover, both instruments have idiosyncrasies that are documented in their individual calibrations, but not with respect to each other. Here, we take advantage of concurrent observations by MapCam and OVIRS to perform a comparison of the datasets. A quantitative comparison enables more in-depth studies of Bennu’s surface variation, taking advantage of the corresponding capabilities of the two instruments to perform high spatial and spectral resolution analyses.

### The OSIRIS-REx Camera Suite

OCAMS is a suite of three scientific imagers designed with individual and overlapping capabilities (Golish et al. 2020; Rizk et al. 2018). PolyCam is a narrow-angle panchromatic camera used to create high-resolution global and regional maps of Bennu’s surface. MapCam is a medium-angle camera with a series of optical filters used to make color maps of Bennu’s surface. SamCam is a moderately wide-angle, panchromatic camera used during and after the sampling event. For the purposes of the comparison with OVIRS, we considered only MapCam. Though the wavelengths imaged by the panchromatic filters in PolyCam and SamCam overlap with OVIRS’s spectral sensitivity, the bandwidth of those filters (∼0.300 μm) is sufficiently broad that a consistent radiometric calibration is challenging for either instrument. More importantly, the color radiometric comparison is more relevant for most analyses that might combine data from both instruments to achieve high spatial and spectral resolution.

MapCam has four narrowband color filters and one wideband panchromatic (pan) filter. The effective wavelengths of the filters are 0.473, 0.550, 0.698, 0.847 and 0.646 μm for the b′, v, w, x, and pan filters, respectively (Golish et al. 2020). The filter cut-on/off wavelengths are 0.439–0.500, 0.521–0.578, 0.671–0.731, 0.815–0.893, and 0.489–0.815, respectively. These filters are comparable to the Eight Color Asteroid Survey bands (Zellner et al. 1985) and were selected to capture spatially resolved variations in Bennu’s spectral slope and band ratios in the visible wavelengths (DellaGiustina et al. 2018).

Additional effort was put into radiometric calibration of MapCam during ground and in-flight calibration due to the sensitivity of color and color ratio mapping of planetary surfaces (DellaGiustina et al. 2020). The calibration effort (Golish et al. 2020) utilized images of Earth’s Moon acquired during the OSIRIS-REx Earth gravity assist (Lauretta et al. 2018) and a Robotic Lunar Observatory (ROLO; Buratti et al. 2011) model of lunar albedo and photometry. Unfortunately, the Moon presented a small target in MapCam’s field of view (∼40 pixels across) and did not provide strong statistics for the calibration. Moreover, MapCam imaged the Moon at a very different sub-spacecraft latitude and longitude than ROLO (which observes from Earth). We applied photometric corrections to the ROLO data to match the conditions of MapCam’s observation, but that process is also very sensitive to the resolution of the image. As a result, the lunar calibration predicted a moderate absolute radiometric uncertainty (±5%, 1$\sigma $). However, MapCam’s four filters share that absolute uncertainty, such that the calibration estimated a low relative (filter-to-filter) radiometric uncertainty of <2%.

The OCAMS imagers have a number of second-order effects that can increase the uncertainty of the radiometric measurements, depending on the conditions of the observations. The OCAMS calibration pipeline does not correct detector non-linearity. The OCAMS detectors are >99.5% linear over most of their dynamic range, but become increasingly non-linear when measuring very high or very low signals (Golish et al. 2020). Nearly all OCAMS images were acquired with exposure times that captured the bulk of the surface within the linear regime. However, extremely bright exogenic material (DellaGiustina et al. 2021) and deep shadows were sometimes imaged with non-linearity greater than 2%.

All OCAMS detectors experience artifacts referred to as *icicles* in images acquired with extremely low exposure times (<3 ms; Golish et al. 2020). OCAMS only acquired images with these exposure times when longer exposures would overexpose portions of the surface. This occurred only for the panchromatic filters of MapCam and PolyCam at low phase angles. For the purposes of this study, icicles were only present for images acquired at 12:30 pm local solar time (Sect. 1.4) with MapCam’s pan filter.

MapCam also has some out-of-field stray light that couples to the detector (Rizk et al. 2018). The stray light is primarily noticeable when there is a bright source just outside MapCam’s field of view, such as when Bennu is larger than the field of view. The noise due to stray light is <1% and is not significant in single-filter images and mosaics, which typically have a signal-to-noise ratio of <1% (DellaGiustina et al. 2020). However, the amount of stray light is wavelength-dependent. Therefore, 0.5% variations due to stray light can add significant noise when calculating color ratios (DellaGiustina et al. 2020), which measure variations on the order of a few percent.

### The OSIRIS-REx Visible and InfraRed Spectrometer

OVIRS is a point spectrometer with a field of view of 4 mrad and a spectral range of 0.4 to 4.3 μm; the full spectrum is obtained simultaneously for each 4 mrad spot (Reuter et al. 2018). OVIRS achieves this spectral range with a series of wedged filters that split five overlapping segments of the full spectral range onto different regions of a Teledyne H1RG infrared detector. The detector is cooled with a passive radiator to reduce dark current and the optics are thermally isolated from the spacecraft deck (Reuter et al. 2018). The first two segments (1a from 0.392–0.670 and 1b from 0.652–1.090 μm) overlap MapCam’s color filters. Importantly, the OVIRS segments image to different locations on the detector in the following order: 1a, 2, 3, 4, 1b. As a result, the two short-wavelength segments are on opposite ends of the detector and may image slightly different regions on the surface when the spacecraft is slewing (Simon et al. 2021). In locations with a sharp discontinuity on the surface (e.g., a deep shadow), the two segments can measure substantially different signals. The boundary between the two segments is approximately at the low-wavelength cutoff of MapCam’s w filter, making segment-related artifacts manifest differently when comparing the b′ and v filters with the w and x filters.

The main science objective of the OVIRS instrument was to detect spectral features and spectral variability of the surface (Kaplan et al. 2020; Lauretta et al. 2021; Simon et al. 2020b), requiring high relative (channel to channel) accuracy (2%) and moderate absolute accuracy (5%). OVIRS’s wavelength range was selected to capture Bennu’s overall VIS-NIR spectral slope and detect absorption features due to hydrated minerals (e.g., 0.7 and 2.7 μm) and organic molecules (e.g., between 3.3–3.6 μm) (Reuter et al. 2018). The OVIRS ground calibration was performed during environmental testing with NIST-traceable sources and showed excellent relative (channel to channel) radiometric accuracy and precision (<1%; Simon et al. 2018, 2021). However, the ground equipment did not cover all wavelengths, and post-testing issues were found with the short wavelength source (Simon et al. 2018, 2021). Data of the Earth were used to adjust the wavelength and radiometric calibration in flight; however, the available dark ocean views were not ideal for cross-calibration with Earth-viewing satellites (Simon et al. 2018). Final adjustments to the radiometric calibration were made using the asteroid itself, based on Earth-based reflectance data, improving calibration in the 2 to 2.5-micron region, but leaving the absolute radiometric accuracy less well defined (>5%).

Additionally, the OVIRS radiometric uncertainty increases when the OVIRS detector is outside its nominal temperature design range (90–105 K), because the detector loses long wavelength sensitivity at higher temperatures, making out-of-band filter effects at all wavelengths more difficult to characterize. This thermal effect was a minor issue in global imaging campaigns, where the detector maintained a temperature around 105 K, primarily due to parasitic heat from the spacecraft itself (Kaplan et al. 2020; Simon et al. 2020b). When the spacecraft was closer to Bennu, however, radiator views of the hot surface caused an increase in the OVIRS detector temperature, increasing the radiometric uncertainty (Simon et al. 2021).

### Scientific Advances Made Possible by Instrument Comparison

Much scientific progress has already been enabled by using MapCam and OVIRS data together. The high spatial resolution of MapCam color images provides a guide for interpreting the geologic context of OVIRS data, whose spectrometer spot size is ∼60× larger than the MapCam pixel scale. Additionally, the broader wavelength range provided by OVIRS can be used to definitively link MapCam color signatures to compositional units, thereby extending the spatial scale where we can discern composition on Bennu. Concurrent observations by MapCam and OVIRS that reveal the same phenomena independently confirm one another. Because of these complementary aspects, examining MapCam and OVIRS in concert can result in substantially more robust scientific interpretations. Below we highlight some major findings made by analyzing data from both instruments in tandem.

The earliest resolved low-phase angle (∼5°) MapCam images of Bennu revealed that Roc Saxum – the largest and darkest exposed boulder on Bennu’s surface – had a shallow absorption feature in the v-band (0.55 μm), consistent with the iron-oxide magnetite (Lauretta et al. 2019a). However, the low spectral resolution of the MapCam colors rendered this interpretation ambiguous. In later MapCam images acquired at higher phase angles (∼8–11°), this absorption feature appeared more shallow, further complicating this interpretation. It was unclear if this change was related to instrumental artifacts or known phase angle effects that can decrease absorption feature depths (e.g., Takir et al. 2015). However, later OVIRS data confirmed the presence of a broad feature centered near 0.55 μm in spectra that are redder than average; the data also revealed two minor lines at 0.50 μm and 0.59 μm (Simon et al. 2020a). Features in this region are usually attributed to an iron transition band (Izawa et al. 2019) and are consistent with the iron oxides magnetite, goethite, and some Fe-bearing phyllosilicates (Cloutis et al. 2011b,a; Sherman and Waite 1985). Of the minerals typically found in aqueously altered carbonaceous meteorites, magnetite is the best spectral match for a 0.55-μm feature with more minor features at 0.50 and 0.59 μm (Simon et al. 2020a). Collectively, the detection of magnetite in MapCam color and OVIRS spectra indicates that Bennu’s parent body underwent extensive aqueous alteration. Examining MapCam data at finer spatial scales (∼25 cm/pixel) indicates that magnetite may be concentrated in dark boulders and freshly exposed surfaces (DellaGiustina et al. 2020).

One of the more surprising discoveries at Bennu was the detection of meter-scale, bright pyroxene boulders on the surface of the asteroid (DellaGiustina et al. 2021). These boulders showed a downturn in the x-band (0.847 μm), the longest wavelength MapCam filter. This downturn is consistent with an absorption feature found in mafic minerals, such as pyroxene or olivine. Since MapCam only captured one shoulder of this presumed absorption, we could make no further inferences on the composition of these boulders. However, spectra collected by OVIRS showed that these bright boulders contained pyroxene and not olivine, as indicated by a second absorption near 2 μm (DellaGiustina et al. 2021). Although Bennu’s blue slope dominated the OVIRS data of these boulders (which occupied ∼1% of the instrument spot size), a pyroxene signature was detected when their spectra were divided by the global average spectrum. These normalized spectra have clear absorption bands at 1 and 2 μm, consistent with calcium-poor pyroxenes. Band centers of the pyroxene absorption bands closely match those in the howardite–eucrite–diogenite meteorites from Vesta and resulted in the conclusion that pyroxene-bearing boulders on Bennu are exogenous (DellaGiustina et al. 2021). This finding has been applied to higher-resolution MapCam data to track the overall distribution of exogenous material on Bennu’s surface (Le Corre et al. 2021; Tatsumi et al. 2021).

Though these studies have examined OVIRS and MapCam data in tandem, the comparisons have mainly been qualitative. In this paper, we summarize the datasets collected by the two instruments and outline recommendations for more accurate, potentially more sensitive comparisons and assessments of uncertainty. Future VIS-NIR studies of Bennu’s mineralogy should use data from both instruments to provide a unified description of the surface at both high spatial and high spectral resolution, following the recommendations we present.

### OSIRIS-REx Observation Campaigns

The OSIRIS-REx mission carried out a series of global and regional imaging campaigns to characterize the surface and potential sample collection sites (Lauretta et al. 2021). OVIRS acquired data in almost every observation campaign; MapCam acquired images in the subset dedicated to color imaging. Table 1 lists the observations used in this work, which are described in detail below.

**Table 1:** OVIRS and MapCam observations used in this comparison

	Date of observation	Average phase angle (°)	Local solar time	Range to surface (km)	Surface coverage
Baseball Diamond					
FB2a	2019 Mar 14	8	12:30 pm	3.6	Global
FB2b	2019 Sep 26	8	12:30 pm	3.6	Global
Equatorial Stations					
EQ1	2019 Apr 25	45	3 pm	5	Global
EQ2	2019 May 02	130	3:20 am	5	Global
EQ3	2019 May 09	8	12:30 pm	5	Global
EQ4	2019 May 16	30	10 am	5	Global
EQ5	2019 May 23	90	6 am	5	Global
EQ6	2019 May 30	130	8:40 pm	5	Global
EQ7	2019 Jun 06	90	6 pm	5	Global
Reconnaissance A					
Sandpiper	2019 Oct 05	35	12:30 pm	1	Regional
Osprey	2019 Oct 12	40	1 pm	0.9	Regional
Kingfisher	2019 Oct 19	40	1:30 pm	1	Regional
Nightingale	2019 Oct 26	30	11:30 am	1	Regional
Reconnaissance B					
Nightingale	2020 Jan 21	65	4 pm	0.65	Regional
Osprey	2020 Feb 11	15	7:30 am	0.7	Regional

The Detailed Survey global imaging mission phase was comprised of the Baseball Diamond and Equatorial Stations campaigns (Lauretta et al. 2021). The Equatorial Stations (EQ) campaign was designed to acquire spectrometer and MapCam data at a series of stations with phase angles ranging from 7° to 130° (Golish et al. 2021a; Lauretta et al. 2021). MapCam and OVIRS acquired all EQ data from the equatorial plane of the asteroid, with a range to surface of approximately 5 km. The spacecraft slewed north/south for at least a full Bennu rotation. For two of the high-phase-angle stations—6 am and 3:20 am (90° and 130° phase, respectively)—the instruments observed for an additional quarter Bennu turn with the spacecraft pointed toward the lit side of the asteroid. OVIRS acquired data in an identical way during all spacecraft slews. MapCam alternated filters every slew, rotating through the full set (pan, b′, v, w, x), such that every fifth slew was imaged with the same filter.

In the Baseball Diamond campaign, OVIRS and MapCam were used concurrently in Flybys 2a (FB2a) and 2b (FB2b). These flybys were designed to acquire MapCam data for color maps of Bennu (DellaGiustina et al. 2020) with a range to surface of ∼3.6 km. FB2b is a re-fly of FB2a, which had large pointing offset to the south caused by a missed spacecraft ephemeris update (Lauretta et al. 2021). Both flybys utilized a point-and-stare observation pattern where MapCam’s pointing was held fixed for all five filters. For FB2b, MapCam acquired images with southern, equatorial, and northern pointings. FB2a had only two pointings and, owing to the missed ephemeris update, the nominally southern and northern looks were pointed off-body and at Bennu’s southern hemisphere, respectively. OVIRS acquired data during the point-and-stares, during the transition between pointings, and from the end of one slew (northern look) to the start of the next (southern look).

After four potential sample sites were selected in the summer of 2019 (Sandpiper, Osprey, Kingfisher, and Nightingale), OSIRIS-REx carried out a series of reconnaissance flybys that imaged the surface at closer ranges (Lauretta et al. 2021). These flybys are referred to as Recon A (∼1 km), Recon B (∼0.62 km), and Recon C (∼0.25 km). Both instruments observed the four candidate sample sites in Recon A; only the final two candidate sample sites (Nightingale and Osprey) were observed in Recon B and Recon C. MapCam acquired images in the Recon A and Recon B campaigns between large PolyCam mosaics, whereas OVIRS acquired data throughout the flyby. As a result, similarly to Baseball Diamond, OVIRS acquired data concurrent with and between groups of MapCam images. Unlike Baseball Diamond, the MapCam data were minimal (sometimes limited to a single set of 10 color images), which limited the time ranges over which comparable OVIRS data were acquired.

## Cross-Instrument Comparison Approach

### Comparison Philosophy

Instrument and observation conditions affected the quality of the acquired data. Both instruments’ calibration pipelines mitigated these effects, but some residual errors were unavoidable without hand-tuned adjustment of individual spectra and images. As such, we approached the comparison of the instruments on a per-dataset basis. That is, we analyzed the relative calibration of the instruments for each set of instrumental and observational conditions independently (e.g., a single Equatorial Station or a single Reconnaissance flyby).

Both instruments have independent absolute radiometric calibrations with moderate uncertainties (Sects. 1.1 and 1.2; Golish et al. 2020; Simon et al. 2018, 2021). The data archived in the Planetary Data System (PDS; Reuter et al. 2019; Rizk et al. 2019) have been calibrated by these published methods, therefore we find it most appropriate to compare the archived calibrated data, rather than attempt to implement an absolute correction. To the notable extent that the absolute radiometric calibrations were different, we did not attempt to determine which instrument was more correct. We established the difference in a rigorous way, and across multiple datasets, to provide future users of these data with context and uncertainties for their analyses.

We performed this analysis using the SPICE kernels (Acton et al. 2018) produced by the OSIRIS-REx navigation team and archived with the Navigation and Ancillary Information Facility (NAIF). Though multiple other analyses, particularly for OCAMS data, have updated the pointing and/or position of MapCam during an observation (e.g., DellaGiustina et al. 2020; Golish et al. 2021b), those updates do not necessarily apply to OVIRS. Registration of the data with Bennu’s shape model has no impact on our results, as the comparison is between instruments. The only impact such alignment had was for creating maps of the comparison (Sect. 4.2), but that impact is less than an OVIRS footprint. Moreover, future users of these data are most likely to characterize them with the kernels available from NAIF. Therefore, it is most broadly applicable to compare the data using the publicly accessible kernels. Nonetheless, using the NAIF kernels for both instruments obfuscated some geometric offset between the two. The SPICE frame and instrument kernels that define the boresights of the instruments were designed in ground testing and updated after launch, but have some residual error. We estimate that the pointing offset between the two instruments was less than an OVIRS footprint in the global imaging campaigns, but likely introduced some error into this analysis (Sect. 4.3).

For a given OVIRS spectrum, we used the MapCam image acquired closest in time for comparison. This minimized the photometric variation that occurs between data acquired at different times, owing either to a change in spacecraft position or to Bennu rotation. OVIRS observations typically started before, and ended after, MapCam imaging. To avoid unbound photometric changes, we limited the OVIRS spectra to those taken between the first and last MapCam images acquired. Even with this constraint, some photometric variation was unavoidable between the OVIRS and MapCam data. In the Equatorial Stations data, MapCam switched filters every slew, repeating every five slews. This results in a *slew aliasing* effect, wherein a given OVIRS spectrum was between zero and two spacecraft slews away from the closest MapCam image with a given filter. The spacecraft completed a slew every 2.7–3° of Bennu rotation, such that the alignment between OVIRS and MapCam data varies between 0 and 6° of Bennu rotation. This had minimal impact at low phase angles (e.g., EQ3), but increasingly large impact at higher phase angles, making these data less reliable.

For the Baseball Diamond flybys, OVIRS data acquired during MapCam’s point-and-stare are temporally aligned, but OVIRS data taken in between MapCam imaging sets and between slews have an offset due to the time gap.

MapCam acquired only sparse data during the Reconnaissance phases, typically only taking one set of images. Thus, we expanded the time window for the Reconnaissance data to include a full scan of the site with OVIRS before and after MapCam imaging. This relaxation increased the amount of data available, but also increased the photometric variation between the data from each instrument significantly.

We further determined data validity by a number of observational factors. OVIRS spectra that were acquired above 50° N/S latitude were excluded, because the high emission angles cause increased uncertainty in the OVIRS radiometric calibration. We removed this limitation for data acquired at the Nightingale site (which is at ∼56°N) in the Reconnaissance phases. We excluded OVIRS spectra with segment discontinuities greater than 2% (Sect. 1.2, 4.1). We excluded panchromatic MapCam images acquired with very short exposures times in EQ3, FB2a, and FB2b (which have icicle artifacts), as well as off-body or calibration MapCam images. Pixels within a MapCam image that were outside the detector’s linear regime (Golish et al. 2020) were also excluded.

### OVIRS Spatial Footprint

**Fig. 1:**
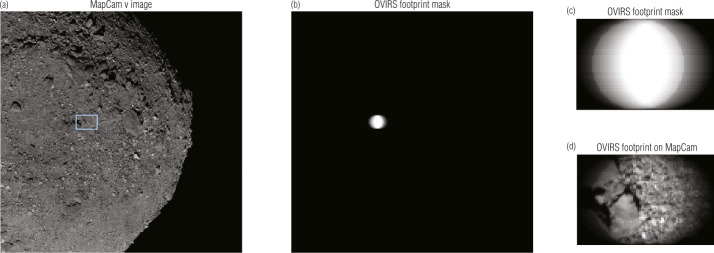
An OVIRS spatial footprint on a MapCam image acquired at 17:53:34 on 2019 May 16. We compared OVIRS spectra to the closest (in time) MapCam image (**a**). The surface locations observed by OVIRS were identified with a weighted mask (**b**, **c**). Taking the mean of MapCam pixels weighted by the mask (**d**; location indicated by the blue rectangle in **a**), for each of MapCam’s filters, produces an equivalent MapCam measurement

For a single OVIRS spectrum, we identified the five images, one for each MapCam filter, acquired closest in time. OSIRIS-REx typically acquired data while the spacecraft slewed and always while Bennu was rotating. For MapCam, the exposure times are short enough that motion blur is $\ll 1$ MapCam pixel. OVIRS’s exposure times, however, typically smeared the OVIRS observation by ∼1/2 of an OVIRS footprint. To account for the changing surface, we calculated the location of the footprint throughout the observation (Fig. 1). To start, we calculated the Bennu latitude and longitude intersected by the OVIRS boresight at the start of the observation, using SPICE kernels and a global shape model (Barnouin et al. 2020; Daly et al. 2020). We calculated latitude and longitude backplanes for every MapCam image and found the pixel in the nearest MapCam image that corresponded to the latitude and longitude of the OVIRS footprint. OVIRS’s field of view is 4 mrad; MapCam’s instantaneous field of view (iFOV; the angle subtended by a single pixel) is 0.067 mrad. Therefore, the OVIRS footprint encompassed pixels within a 59-pixel diameter of the center point. We then translated the OVIRS footprint from the start to the end of that OVIRS observation. At 100 points along the track, we repeated the footprint calculation, building a weighted OVIRS mask (Fig. 1(c)). The center of the track was more heavily weighted because OVIRS observed it throughout the integration, whereas it observed the edges only at the beginning or end. We applied this mask to the MapCam image to calculate a weighted average of MapCam pixels corresponding to this OVIRS spectrum. We then took the mean of those pixels, because a mean represents OVIRS’s physical averaging of photons from multiple surface locations. We repeated this for each MapCam filter to produce a five-point MapCam spectrum corresponding to the OVIRS spectrum.

### MapCam Spectral Footprint

**Fig. 2:**
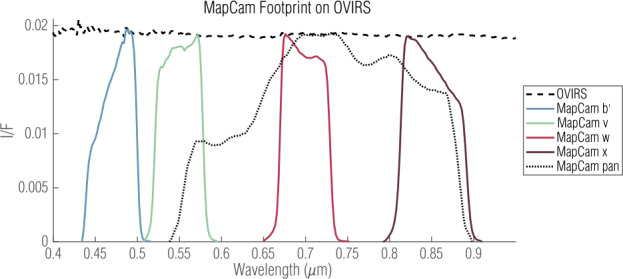
MapCam spectral footprints, from ground-based responsivity testing, plotted with an example OVIRS spectrum acquired at 17:53:46 on 2019 May 16

Similarly, we extracted a five-point OVIRS spectrum from an OVIRS observation by imparting a MapCam spectral footprint. MapCam’s spectral responsivity was characterized in extensive ground testing and documented in Golish et al. (2020). The per-filter spectral responsivity included filter transmission, optics throughput, and detector sensitivity. We multiplied an example OVIRS spectrum (Fig. 2) by the normalized MapCam responsivities to produce a five-point OVIRS spectrum.

### Spectral Comparison

**Fig. 3:**
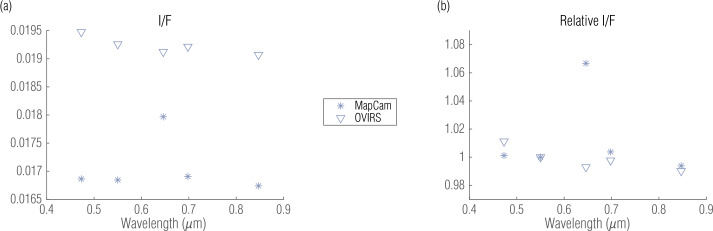
Five-point spectral ratios for the example shown in Sects. 2.2/2.3, in absolute (**a**) and relative (**b**) terms, compares the response of each instrument at the same location (∼23S, 272E) on Bennu’s surface

For each OVIRS spectrum, we calculated the ratio of the five-point spectra in both absolute and relative terms. The absolute five-point ratio (Fig. 3(a)) gives the absolute radiometric offset between the two instruments for each of MapCam’s filters. The relative five-point ratio (Fig. 3(b)), which we normalized to the v filter (0.55 μm), expresses the filter-to-filter offset of the two instruments. We are primarily interested in how the four narrowband MapCam filters compare with OVIRS. Though the pan filter also overlaps OVIRS wavelengths, it is not as useful for spectral comparison because of the width of the filter. Nonetheless, we included it in the analysis for completeness.

**Fig. 4:**
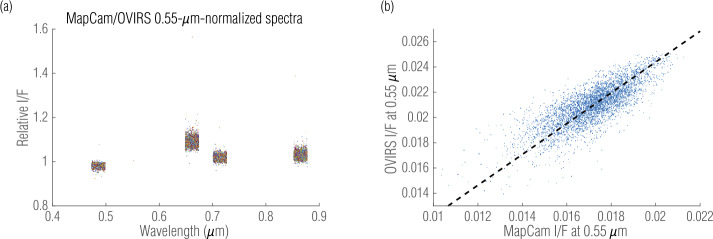
Five-point spectra normalized to MapCam’s v filter (0.55 μm) illustrate the mean and variation of the offset between the instruments (**a**). Individual points are colored arbitrarily and randomly spread over 50 μm, around the filter’s center wavelength, to help distinguish individual points among the cluster. Plotting the measured I/Fs against each other (**b**) further illustrates the comparison, where the dashed line has a slope of 0.82 (equivalent to the absolute radiometric offset between the two instruments; see Sect. 3.1) and scatter around that line is indicative of variation between the instruments

We repeated this comparison for every valid OVIRS spectrum. We depict the mean and variation of each filter by plotting the relative ratios on a scatter plot—with small, random perturbations in wavelength for visualization (Fig. 4(a)). Here the spectra are normalized to the v filter; therefore, all v-filter data have a mean of exactly 1 with no variation. We also plotted the reduced I/F from both instruments (Fig. 4(b); v filter). If the instruments were perfectly calibrated, the data would fall on the 1:1 dashed line. To the extent that their absolute radiometric calibration differs (Sects. 1.1 and 1.2; Fig. 3(a)), the data would fall along a line with a different slope. Because the data were noisy, they populate a scatter envelope around the line. These results, for each OVIRS spectrum, MapCam filter, and OSIRIS-REx observation campaign, were compiled to produce a per-filter, per-dataset comparison of the two instruments.

## Cross-Instrument Comparison Results

### Global Observation Campaigns

**Fig. 5:**
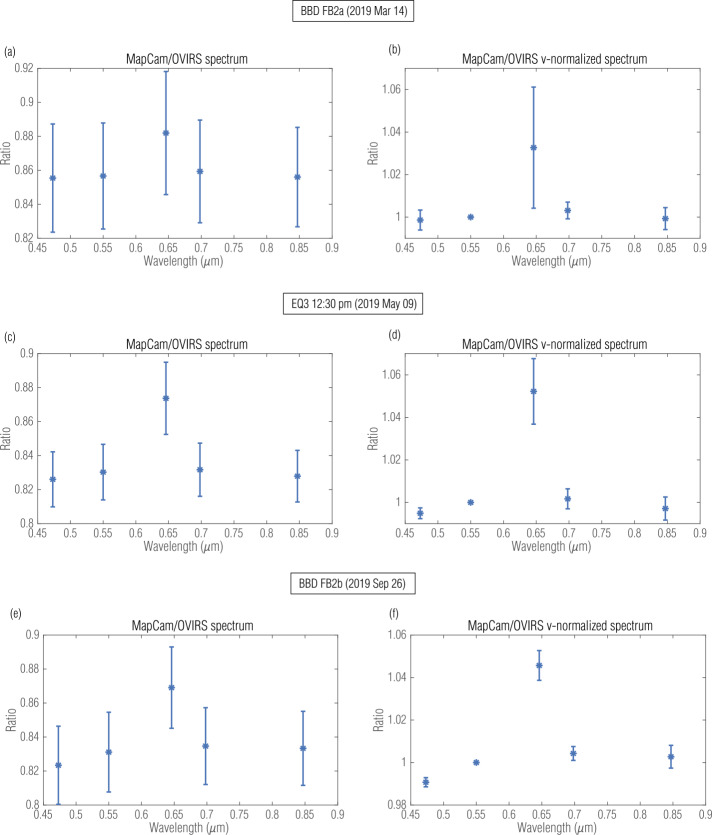
Median five-point absolute (left; Table 2) and relative (right; Table 3) spectral ratios for FB2a, EQ3, and FB2b data ($12:30$ pm LST, ∼8° phase angle)

The global observation campaigns provided the best opportunity to compare the instruments, particularly those acquired at low phase angle. The FB2a, EQ3, and FB2b datasets were all acquired at 12:30 pm local time (∼8° phase angle). These low-phase-angle observations had minimal shadows, which are otherwise prevalent on Bennu’s rough surface. Following the procedure described above, we calculated the per-filter median and standard deviation of all five-point absolute and relative spectral ratios (Fig. 5). MapCam’s pan filter had a ∼12% absolute radiometric offset from OVIRS in these data, despite the fact that the pan filter essentially encompasses MapCam’s v and w filters. The difference was a result of the MapCam radiometric calibration (Golish et al. 2020), which noted a higher response by the pan filter than the other filters. The narrowband filters all had absolute offsets between 17 and 18%, suggesting a large discrepancy between the MapCam and OVIRS (Table 2). However, when normalizing to the v filter at 550 nm (i.e., removing the absolute offset), the four narrowband filters compared very well to OVIRS (<1% residual offset; Table 3). This suggests that any comparative analysis that uses color ratios (OVIRS to MapCam or MapCam filter to filter) would have radiometric uncertainty of <1%.

**Table 2:** Absolute I/F ratios from cross-instrument global comparisons

	Median absolute MapCam/OVIRS ratio (±1*σ*)				
	b′	v	w	x	pan
EQ3 (12:30 pm)	0.826 ± 0.016	0.830 ± 0.016	0.832 ± 0.016	0.828 ± 0.015	0.874 ± 0.021
FB2a (12:30 pm)	0.855 ± 0.032	0.857 ± 0.031	0.859 ± 0.030	0.856 ± 0.029	0.882 ± 0.036
FB2b (12:30 pm)	0.823 ± 0.023	0.831 ± 0.023	0.835 ± 0.023	0.833 ± 0.022	0.869 ± 0.024
EQ4 (10 am)	0.801 ± 0.046	0.818 ± 0.045	0.829 ± 0.043	0.837 ± 0.041	0.885 ± 0.043
EQ1 (3 pm)	0.854 ± 0.082	0.828 ± 0.083	0.796 ± 0.083	0.765 ± 0.085	0.766 ± 0.094
EQ5 (6 am)	0.766 ± 0.111	0.794 ± 0.115	0.810 ± 0.119	0.830 ± 0.129	0.876 ± 0.151
EQ2 (3:20 am)	0.648 ± 0.481	0.754 ± 0.522	0.852 ± 0.499	0.905 ± 0.496	0.990 ± 0.594

**Table 3:** Normalized I/F ratios from cross-instrument global comparisons

	Median v-normalized MapCam/OVIRS ratio (± 1*σ*)				
	b′	v	w	x	pan
EQ3 (12:30 pm)	0.995 ± 0.003	1	1.002 ± 0.005	0.997 ± 0.005	1.052 ± 0.015
FB2a (12:30 pm)	0.999 ± 0.005	1	1.003 ± 0.004	0.999 ± 0.005	1.033 ± 0.029
FB2b (12:30 pm)	0.991 ± 0.002	1	1.004 ± 0.003	1.003 ± 0.005	1.046 ± 0.007
EQ4 (10 am)	0.979 ± 0.007	1	1.014 ± 0.010	1.024 ± 0.014	1.083 ± 0.018
EQ1 (3 pm)	1.033 ± 0.014	1	0.959 ± 0.017	0.921 ± 0.027	0.920 ± 0.042
EQ5 (6 am)	0.962 ± 0.032	1	1.022 ± 0.036	1.050 ± 0.066	1.113 ± 0.106
EQ2 (3:20 am)	0.799 ± 0.195	1	1.221 ± 0.293	1.390 ± 0.623	1.653 ± 1.080

Data from FB2a and FB2b had similar absolute and relative offsets (Fig. 5(a,b,e,f), Table 2), though with slightly higher standard deviations (represented as error bars). As noted in Sect. 1.4, the Equatorial Stations and Baseball Diamond flybys had different types of slew aliasing, which likely introduced photometric differences between the observations that increased the noise. Moreover, OVIRS did not have complete surface coverage in Baseball Diamond (see maps in Sect. 4.2) owing to the MapCam-driven observation strategy. FB2a’s absolute offset was slightly less (∼14%), despite having nearly identical imaging geometry to FB2b. However, OVIRS data in FB2a were slightly saturated over some brighter portions of the surface. This would result in depressing the reflectance that OVIRS measured, thereby decreasing the offset with respect to OCAMS. Nonetheless, the relative offset remained <1% for all three low-phase global datasets (Table 3).

**Fig. 6:**
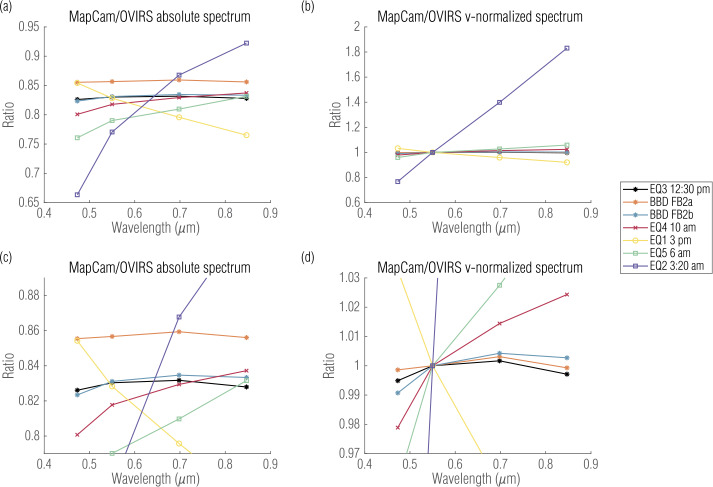
Absolute (**a**, **c**) and relative (**b**, **d**) ratios comparing OVIRS with MapCam’s narrowband filters for the global datasets. Figures (**c**) and (**d**) plot the same data as (**a**) and (**b**), respectively, with a cropped y-axis to better visualize the comparison of the best datasets

As phase angle increased in the other Equatorial Stations, so did the shadows on the surface, which in turn increased the offset and noise between the two instruments. The data collected during EQ4 (10 am, ∼30° phase), for example, still compared well (∼2% variation between instruments in the v-normalized spectrum; Table 3), with a slightly higher absolute offset (∼20%; Table 2). However, the higher-phase stations became increasingly variable. Figure 6 plots the absolute and relative ratios for each of the global datasets on the same axes. If the instruments were perfectly calibrated, with respect to each other, these ratio spectra would be horizontal lines with a ratio value of 1. Deviations from a value of 1 indicate a calibration offset between OVIRS and MapCam for that dataset. These ratio spectra highlight that the low-phase-angle data have smaller offsets and compare better with each other than with higher-phase-angle data.

OSIRIS-REx was pointed toward the terminator and the night side of Bennu during the 6 pm and 8:40 pm equatorial stations, respectively. As a result, both the OCAMS and OVIRS five-point spectra measure primarily noise and are not included here. The same was true for most of the 6 am and 3:20 am equatorial stations, but OSIRIS-REx acquired data for a quarter-Bennu-turn with the spacecraft pointed toward the lit side of the asteroid. The data from just the quarter-turn were included here, but were quite noisy, leading to larger offsets, particularly for 3:20 am. The 3 pm station had not only larger offsets, but also a different spectral trend than the other stations. Although at these phase angles it is difficult to assign a cause definitively, the direction of shadows likely played a role. Because we excluded the 6 pm and 8:40 pm stations, the 3 pm station was the only one analyzed here with eastward shadows (the other stations were in the morning or close to noon). Shifting the shadows may change the instruments’ relative response to the surface, considering any pointing offset between them and the OVIRS segment read-out order mentioned in Sect. 1.2. We tabulate the median and standard deviation, per dataset, of the absolute MapCam/OVIRS ratios in Table 2 and of the relative spectra in Table 3.

### Regional Observation Campaigns

Comparing the instruments during the OSIRIS-REx Reconnaissance campaigns was more challenging due to the closer range to the surface, which increased the OVIRS detector temperature, noise, and radiometric uncertainty. This environment directly affected the absolute radiometric ratio of the two instruments, but was less impactful on the relative ratio. Increased OVIRS detector temperature decreases the long-wavelength sensitivity (Simon et al. 2018, 2021), which is outside MapCam’s spectral coverage. Higher temperatures can affect the correction of out-of-band leaks at short wavelengths (Simon et al. 2021), but we mitigated this by excluding spectra with large discontinuities (Sects. 2.1, 4.1).

**Fig. 7:**
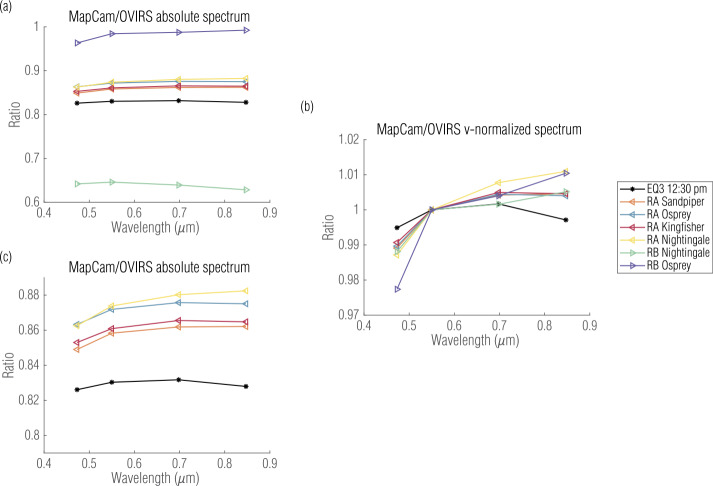
Absolute (**a**, **c**) and relative (**b**) ratios comparing OVIRS with MapCam’s narrowband filters for each of the regional datasets (Recon A and B, abbreviated RA and RB), shown with the EQ3 dataset for comparison. Figure (**c**) plots the same data as (**a**), with a cropped y-axis to better visualize the comparison of the best datasets

The closer range also amplified the effect of the instruments’ angular pointing inaccuracies. These inaccuracies were much less than an OVIRS footprint when the spacecraft was 3.6–5 km from the surface, but the inaccuracies increased linearly with decreased distance. At ranges of ∼1 km (Recon A) and ∼0.62 km (Recon B), the pointing offset was a significant fraction of an OVIRS footprint. This caused increased differences between individual OVIRS spectra and their corresponding OCAMS footprint. On the other hand, the regional nature of the data decreased the scatter induced by varying albedo on Bennu. As a result, the relative radiometric ratios for the regional datasets were only 2–3% (Fig. 7), but had standard deviations several times larger (Table 4). Again, for perfectly calibrated instruments, these median ratios would be 1 at all wavelengths. However, these regional datasets emphasize that both the median ratio, and standard deviation around that median, are needed to represent the fidelity of the cross-instrument comparison.

**Table 4:** Absolute I/F ratios from cross-instrument Recon A ($RA$) and Recon B ($RB$) comparisons

	Median absolute MapCam/OVIRS ratio (±1*σ*)				
	b′	v	w	x	pan
Sandpiper (RA)	0.849 ± 0.070	0.858 ± 0.071	0.862 ± 0.069	0.862 ± 0.069	0.882 ± 0.071
Osprey (RA)	0.863 ± 0.121	0.872 ± 0.124	0.876 ± 0.120	0.875 ± 0.121	0.892 ± 0.126
Kingfisher (RA)	0.853 ± 0.074	0.861 ± 0.075	0.866 ± 0.073	0.865 ± 0.072	0.883 ± 0.077
Nightingale (RA)	0.863 ± 0.297	0.874 ± 0.296	0.880 ± 0.291	0.882 ± 0.287	0.901 ± 0.302
Nightingale (RB)	0.642 ± 0.594	0.646 ± 0.593	0.639 ± 0.582	0.628 ± 0.551	0.657 ± 0.584
Osprey (RB)	0.963 ± 0.269	0.984 ± 0.276	0.988 ± 0.276	0.992 ± 0.283	1.054 ± 0.307

## Comparison of Individual Spectra

The combined results from the previous sections demonstrate reasonably good agreement between the two instruments when averaged over entire datasets from discrete observational campaigns. However, the standard deviations attached to those averages (which are performed over thousands of spectra) indicate significant spectrum-to-spectrum variation. In general, the variations (both filter-to-filter and as represented by 1$\sigma $ error bars) listed in the previous section should be used as uncertainties for any cross-instrument comparison that uses individual spectra (Table 2 and Table 4 for absolute comparisons, Table 3 and Table 5 for filter-relative comparisons). The differences between the instruments discussed in Sect. 1 have a direct impact on the comparison of individual spectra.

**Table 5:** Normalized I/F ratios from cross-instrument Recon A ($RA$) and Recon B ($RB$) comparisons

	Median v-normalized MapCam/OVIRS ratio (±1*σ*)				
	b′	v	w	x	pan
Sandpiper (RA)	0.989 ± 0.003	1	1.004 ± 0.006	1.005 ± 0.008	1.028 ± 0.010
Osprey (RA)	0.990 ± 0.005	1	1.004 ± 0.009	1.004 ± 0.012	1.024 ± 0.013
Kingfisher (RA)	0.991 ± 0.004	1	1.005 ± 0.009	1.005 ± 0.014	1.025 ± 0.016
Nightingale (RA)	0.987 ± 0.004	1	1.008 ± 0.009	1.011 ± 0.011	1.031 ± 0.008
Nightingale (RB)	0.988 ± 0.027	1	1.002 ± 0.039	1.005 ± 0.064	1.026 ± 0.031
Osprey (RB)	0.977 ± 0.019	1	1.004 ± 0.018	1.010 ± 0.031	1.071 ± 0.056

### Segment Discontinuities

**Fig. 8:**
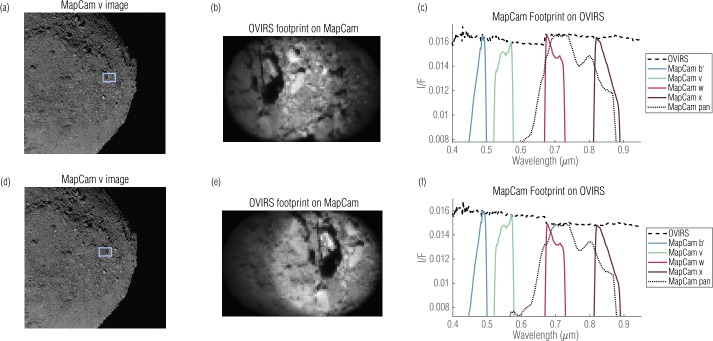
Large shadows on the surface observed by MapCam (**a**, **d**; 17:53:42 on 2019 May 16)—such as one located at ∼43°S, 272°E (**b**, **e**; location indicated by the blue rectangle in **a**, **d**)—are more susceptible to segment discontinuities in OVIRS spectra (**c**, **f**, dashed black lines; 17:53:38 and 17:53:40 on 2019 May 16), due to the 1a and 1b segments imaging portions of the surface with different brightness. MapCam filters (**c**, **f**, color lines) are sensitive to wavelengths on either side of the discontinuity

As discussed in Sect. 1.2, OVIRS has a segment boundary at approximately the short-wavelength end of the OCAMS w filter. Because the two segments that compose this boundary are on opposite sides of the OVIRS detector, and read out at slightly different times, they imaged slightly different portions of the surface as the spacecraft was slewing during an OVIRS integration. If these portions of the surface were not spatially uniform, the two segments could have measured signals that were different in proportion to that heterogeneity. Figure 8 depicts an example of this where OVIRS observed a large shadow in two subsequent integrations. In the first spectrum (Fig. 8(a-c)), the shadow strongly influenced segment 1a and suppressed the signal below ∼0.68 μm. In the second spectrum (Fig. 8(d-f)), the spacecraft had slewed such that segment 1b was most affected by the shadow, suppressing the longer wavelengths. In the analyses described above, we rejected any OVIRS spectrum with a segment discontinuity larger than 2%. Continuity was calculated by taking the median of the spectra over the wavelengths 0.040 μm before and after the boundary (i.e., 0.64–0.68 μm and 0.68–0.72 μm). However, such discontinuities can influence individual spectra for analyses of specific surface features that were much brighter or darker than their surroundings.

### Maps

To better visualize the spatial distribution of differences between the two instruments, we produced maps of the OVIRS and MapCam comparisons. We constructed these maps by averaging OVIRS footprints into latitude/longitude bins. As such, the maps are at OVIRS’s approximate spatial resolution. Though this approach sacrificed MapCam’s much finer spatial resolution, it maintained a 1:1 spatial match between the two datasets (as opposed to comparing the OVIRS map with a native resolution MapCam mosaic such as those in DellaGiustina et al. (2020)). These maps facilitate the comparisons of various albedo or spectral parameter maps derived from the two instruments (e.g., DellaGiustina et al. 2020; Fornasier et al. 2020; Golish et al. 2021b; Kaplan et al. 2020; Li et al. 2021; Simon et al. 2020; Zou et al. 2021).

**Fig. 9:**
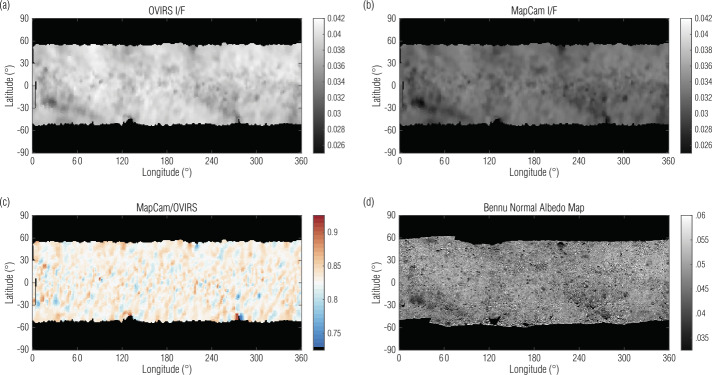
Comparisons of OVIRS (**a**) and MapCam (**b**) EQ3 I/F maps illustrate the absolute offset between the instruments. A ratio of the two I/F maps (**c**) shows the terrain- and slew-dependent noise in the comparison. The Bennu normal albedo map (**d**; Golish et al. 2021c) is included for visual reference, it was not used in the analysis

Figure 9 shows OVIRS, MapCam, and ratio maps for the EQ3 (12:30 pm) dataset. The Bennu albedo map (Golish et al. 2021c) is also shown for reference; it is not used in this analysis. The albedo map values are not directly comparable because the albedo map has been photometrically corrected and the EQ3 I/F maps have not, but there are qualitative spatial correlations between the maps. As shown in the previous section (Fig. 6 and Table 2), OVIRS measured the mean I/F as ∼17.5% larger than MapCam did. As expected, many of the regions that deviated from this mean (higher or lower) corresponded to large features on Bennu’s surface that cast shadows, even at low phase angles. These features often have a ‘bright’ side and a ‘dark’ side in the ratio map, presumably due to slightly different photometric conditions between MapCam and OVIRS as the spacecraft slewed over the features.

The remaining structure is not random and is likely driven by the photometric variation induced by slew aliasing between OVIRS and MapCam data, coupled with Bennu’s terrain. Regardless of its source, this structure will interfere with any individual spectrum comparison. Though the v-normalized spectra, on average, agree within 1% between the two instruments, and with a standard deviation <1% (Fig. 5), comparing individual spectra can have differences as high as 10% around large surface features.

**Fig. 10:**
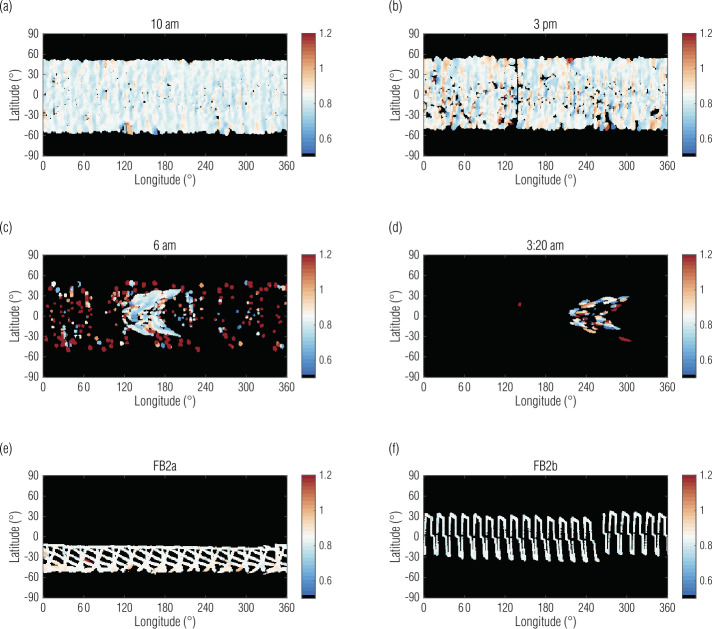
Maps of MapCam/OVIRS ratios where their coverage overlaps in the global campaigns, when compared with EQ3 at 12:30 pm (Fig. 9), illustrate decreasing utility with increasing phase angle. The color scale represents the MapCam/OVIRS ratio. Data with ratios around the instruments’ radiometric offset (∼0.82) compare well between the instruments; data far from that offset indicate poor cross-comparison. The FB2a and 2b maps illustrate the sparse OVIRS coverage during the MapCam-focused flybys

At higher phase angles (and therefore larger shadows), differences induced by terrain and slew aliasing become more pronounced. Even at 10 am (Fig. 10(a)), vertical artifacts resulting from slew aliasing become qualitatively obvious. At 3 pm (Fig. 10(b)), as discussed in Sect. 3.1, shadows were larger and in the opposite direction. At 6 am and 3:20 am (Fig. 10(c,d)), only the lit quarter turn provided usable data, which covered a small portion of the surface and did so with large shadows and resulting noise.

The Baseball Diamond data (FB2a and FB2b) compared well between the two instruments, as we would expect for low phase angles. However, the ratio maps (Fig. 10(e,f)) illustrate the sparse OVIRS coverage during these MapCam-focused observations. As such, the EQ3 data are generally preferred.

**Fig. 11:**
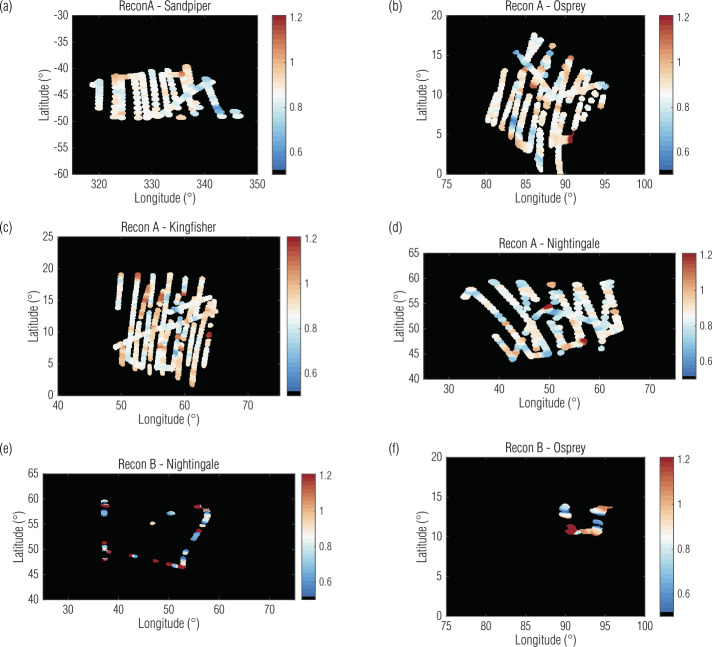
Maps of overlapping MapCam/OVIRS ratios (indicated by the color scale) for the regional campaigns illustrate large variability in the Recon A data when comparing the instruments. Recon B data, which rarely met our data validity requirements, sparsely covered the site and were not reliable in an instrument-to-instrument comparison without further spectrum-specific calibration

The Recon A data had much more variation in ratios, as we would expect for the mid-phase angles and closer range to surface. As shown in Table 4, though the median MapCam/OVIRS ratio is similar to the low phase global campaigns, the standard deviation and the variation in the ratio maps (Fig. 11(a-d)), are much higher. This emphasizes that the instruments were spectrally similar on average but have significant spectrum-to-spectrum variation. Any analysis that includes individual spectrum comparisons should acknowledge this variation. Finally, the maps for Recon B Fig. 11(e,f) illustrate the lack of utility of these data. The data that passed even our relaxed validity constraints (Sect. 2.1) were noisy and did not cover the bulk of the sampling sites. We include the Recon B results not as reliable statistics for future analyses, but as caution against using them without further calibration and analysis.

### Spatial Co-registration

**Fig. 12:**
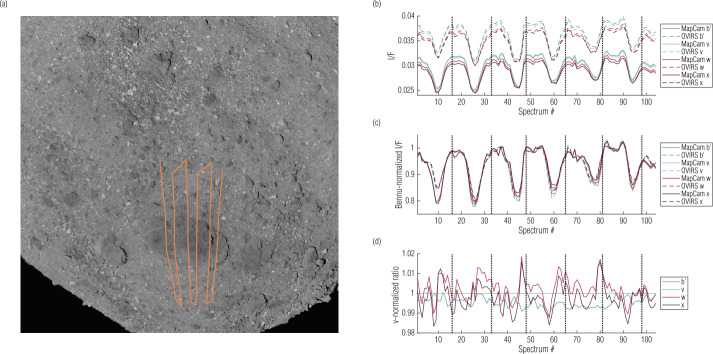
Spectral tracks from OVIRS and MapCam as the instruments slewed over Roc Saxum (**a**). In the full observation, the data continued toward the northern part of Bennu before slewing back over Roc Saxum; we show only a subset of the slews here. Plots of I/F ratios (**b**), Bennu-normalized I/F ratios (**c**), and v-normalized I/F ratios (**d**) track the response of the instruments throughout the slews. Vertical dotted lines indicate the beginning and end of the slews that imaged Roc Saxum

As described in Sect. 2.1, we made no attempt in this analysis to align the OVIRS and MapCam data. The pointing of the data from both instruments is described in the mission kernels derived by the OSIRIS-REx navigation team and archived with NAIF. Nonetheless, the ratio maps shown in the previous section depict reasonable spatial co-registration. We further evaluate that registration by plotting OVIRS and MapCam spectra as the instruments slew over notable surface features. Roc Saxum (∼25° E, 25° S), in Bennu’s southern hemisphere, is ∼20% darker than average Bennu and ∼100 m long, making it the most prominent albedo feature on Bennu’s surface. Figure 12(a) plots the OVIRS observation track as it slewed over Roc Saxum six times during the EQ3 (12:30 pm) station. The absolute I/F tracks (Fig. 12(b)) are indicative of the absolute radiometric offset between the instruments. However, when we normalized the spectral tracks to Bennu’s average I/F (as measured by each instrument and filter), they reveal that the MapCam data undergo a deeper drop in Bennu-normalized I/F in the first slew (Fig. 12(c)). This slew was along the eastern edge of Roc Saxum, which was the shadowed edge because these data were acquired slightly past noon (local solar time). We have seen throughout the analysis that shadows were the biggest driver for differences between the instruments, which seems to be confirmed here. In addition, because the first slew was along a relatively sharp albedo transition, any east-west misregistration between the two instruments would manifest as a difference here.

Figure 12(d) plots the v-normalized spectral ratios for the slews over Roc Saxum, showing several deviations, particularly in the w and x filters. These deviations are most prominent when the instruments slewed on and off Roc Saxum (i.e., coincident with a rapid change in albedo). As described in Sect. 1.2, the w and x filters correspond to the OVIRS segment 1b, which was imaging a slightly different part of the surface than segment 1a (MapCam filters b′ and v). This rolling shutter effect likely results in w- and x-filter deviations. The width of these deviations are a few OVIRS integrations, giving a rough sense of the spatial offset (∼10 m) in this dataset. However, even these outlier spectrum-to-spectrum deviations are less than 2%, while most deviations are less than 1%, indicating reliable comparison between the instruments.

### Sample Spectra

**Fig. 13:**
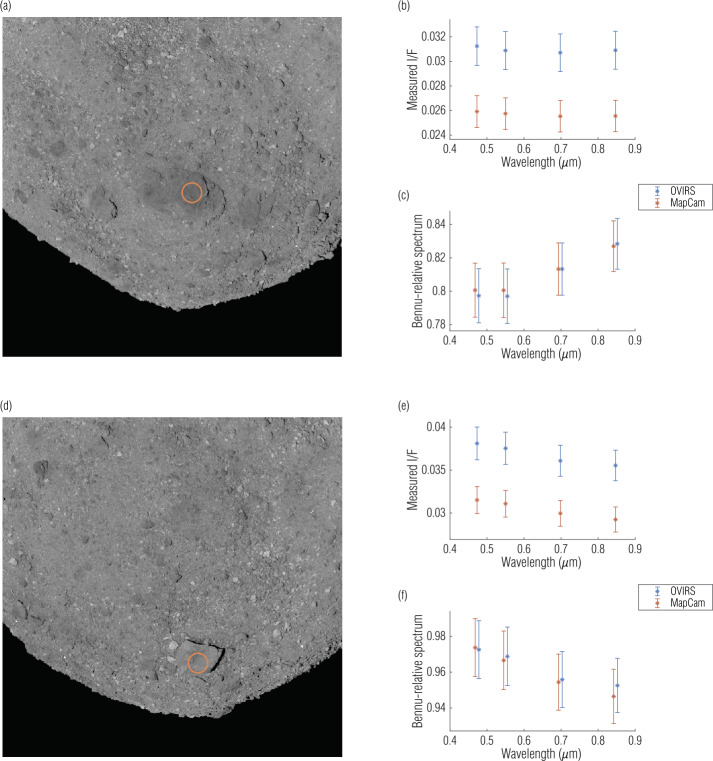
Individual spectra of Roc (**a**) and Benben (**d**) Saxa indicate the validity and uncertainty of OVIRS and MapCam comparisons. The absolute I/F offset (**b**, **e**) is larger than the ∼5% radiometric uncertainty predicted by both instruments (indicated by the error bars). However, when compared filter-to-filter (**c**, **f**), OVIRS and MapCam agree to $<1\%$

Despite the qualifications and uncertainties detailed throughout this analysis, meaningful comparative work can be and has been performed (DellaGiustina et al. 2021; Kaplan et al. 2020) by cross-referencing data from the two instruments. Using two notable surface features—Roc Saxum and another large boulder, Benben Saxum—Fig. 13 plots the absolute and relative spectra acquired by both instruments during EQ3. The relative spectrum was normalized to Bennu’s average spectrum (calculated using the EQ3 data). We selected spectra from the middle of the boulders because data from the edges can lead to artifacts. The spectra from the two instruments have the 17–18% absolute radiometric offset established in Sect. 3.1. However, the Bennu-relative spectra agree within 1% and confirm the colors (DellaGiustina et al. 2020; Simon et al. 2020b) of these features: Roc Saxum and Benben Saxum are redder and bluer, respectively, relative to average Bennu. A filter-relative analysis using these data, or any EQ3 data, should carry the uncertainties identified in Table 3 (±1%).

## Conclusions

This work provides a complete summary of concurrent OVIRS and MapCam datasets acquired during OSIRIS-REx proximity operations and recommendations for how to most accurately compare them.

The instruments have a large absolute radiometric offset (∼15–20%) that stems from independent calibration processes with independent sources of error. However, the offset is consistent among all four MapCam color filters for the low-phase-angle datasets. In low-phase-angle observations, when shadows and instrument effects have minimal impact on the data quality, the OVIRS-to-MapCam and MapCam filter-to-filter relative calibration are very good (<1% uncertainty). The EQ3 dataset (acquired at ∼12:30 pm local solar time) provides the most thorough surface coverage and highest-quality cross-instrument comparison, with a <2% spectrum-to-spectrum 1$\sigma $ absolute radiometric uncertainty.

We strongly recommend using this dataset whenever possible when comparing data from these two instruments, because higher-phase-angle data require larger uncertainties to be applied. Even with the EQ3 dataset, we advise some caution when analyzing individual OVIRS spectra, due to imperfect instrument co-registration and OVIRS segment discontinuities. Nonetheless, this cross-instrument comparison allows future analyses to apply realistic uncertainties to overlays, ratios, and other quantitative comparisons of OVIRS and MapCam data acquired at Bennu, and perhaps to identify subtler signals that have been previously discernable.

## Data Availability

The OVIRS (Reuter et al. 2019) and OCAMS (Rizk et al. 2019) data used in this analysis are available at the Planetary Data System Small Bodies Node (https://sbn.psi.edu/pds/resource/orex/). The results of this analysis—tabulated data from Tables 2 and 3 and raster images representing the ratio maps in Figs. 9, 10, and 11—are archived in Golish (2022).
